# Guidelines (1987) for classification, calculation and validation of conversion rates in clinical chemistry – International Federation of Clinical Chemistry, Scientific Committee, Analytical Section: Expert Panel on Analytical Systems, Expert Panel on Enzymes; IFCC Document Stage 2, Draft 4

**DOI:** 10.1155/S1463924687000245

**Published:** 1987

**Authors:** G. Bechtler, R. Haeckel, M. Hørder, H. Küffer, A. J. Porth

**Affiliations:** Zentralkrankenhaus Institut für Laboratoriumsmedizin Bremen 1 D2800 Germany


Journal of Automatic Chemistry/*Journal of Clinical Laboratory Automation*, Vol. 9, No. 3 (July–September 1987), pp. 105–112

### Guidelines* (1987) for classification, calculation and validation of conversion rates in clinical chemistry

### International Federation of Clinical Chemistry, Scientific Committee, Analytical Section: Expert Panel on Analytical Systems[1], Expert Panel on Enzymes[2]; IFCC Document Stage 2, Draft 4

**Prepared for publication by G. Bechtler (DE), R. Haeckel[3] (DE), M. Hørder (DK), H. Küffer (CH) and A. J. Porth (DE)**

[1] *Members of the Expert Panel on Analytical Systems: C. A. Burtis (USA), Chairman, T. D. Geary (Australia), T. Sasaki (Japan).*
[2] *Members of the Expert Panel on Enzymes: R. Rej (USA), Chairman, M. Hørder (Denmark), M. Mathieu (France), L. Shaw (USA), J. H. Strømme (Norway).*
[3] *To whom enquiries and reprint requests should be addressed: Zentralkrankenhaus, Institut für Laboratoriumsmedizin, D2800 Bremen 1, FR Germany.*

### Contents

1 Introduction
2 Classification of 'conversion rate methods'
2.1 Discontinuous methods
2.2 Continuous methods
3 Theoretical aspects of minimal requirements for evaluation of rates of conversion for calculating catalytic activity concentrations of enzymes
3.1 Current definitions and recommendations by professional societies
3.2 Variable rates of enzyme-catalysed reactions due to the mechanism of enzyme catalysis
3.3 Variable rates of enzyme-catalysed reactions not inherent to the mechanism of catalysis
3.4 Blank reactions
4 Calculation and evaluation methods for the determination of the catalytic activity concentration of enzymes
4.1 Calculation methods
4.1.1 Delta methods
4.1.2 Regression methods
4.1.3 Derivative methods
4.1.4 Integral methods
4.2 Evaluation methods
4.2.1 Visual evaluation
4.2.2 Evaluation of multiple-point procedures
4.2.3 A procedure to determine confidence limits for calculated results
5 Calculation and evaluation methods for the determination of analyte concentration from conversion rates
5.1 Theoretical considerations and calculation methods
5.1.1 Zero-order reactions
5.1.2 First-order reactions
5.1.3 Second-order reactions
5.2 Advantages and disadvantages of conversion rate methods for the determination of analyte concentrations
6 References
7 Key to symbols

© 1987 IFCC

* These guidelines are based on a former version [1] which has been revised to take account of the comments received.

### 1. Introduction

Results from chemical reactions can be calculated by using either kinetic or equilibrium data acquisition. The corresponding procedures are therefore commonly called either kinetic or equilibrium methods, depending on whether measurements are made before or after equilibrium state of the reaction is obtained. Only kinetic methods are considered in the following. Data from the early course of the reaction can also be used to predict the total change in sensor signal at equilibrium from which the concentration of an analyte may be calculated as for an equilibrium procedure [2]. Since the data used for the calculation are measured during the progress of the reaction such a procedure should be classified as kinetic.

Quantitative results are calculated from rates of conversion formerly often called rate of reaction (see section 2, last paragraph) by almost as many procedures as there are different instruments on the market [3]. The nomenclature used by the manufacturers and also in the scientific literature varies considerably and therefore some degree of standardization appears desirable.

The main purpose of the guidelines is to recommend nomenclature and minimal requirements for the calculation of results from rates of conversion. Only those aspects were considered, which appear to have practical relevance at the present time. The document contains sections on classification and theoretical background of rate measurements and two applications, first on the determination of the catalytic activity concentration of enzymes and second on the determination of analyte concentrations from conversion rates.

### 2. Classification of 'conversion rate methods'

The classification proposed distinguishes between those methods where a quantity is measured at different times (discontinuously) and those where it is continuously monitored (table 1). As indicated in the introduction only those procedures are listed in table 1 which have been used in practice [4 and 5]; there are other theoretical possibilities. Measured quantities can be of different kinds such as volumes or spectrometric or electrical quantities. Therefore, the considerations discussed in this recommendation are not restricted to spectrometric measurements (although the examples given refer mainly to spectrometric methods).

*Table 1. Classification of conversion rate methods used in clinical chemistry.*

| | |
|---|---|
| 1. | Discontinuously monitoring procedures |
| 1.1 | One point procedures for determining rate of conversion |
| 1.1.1 | with stopped reaction |
| 1.1.2 | with reaction in progress |
| 1.2 | Two point procedures for determining rate of conversion |
| 1.2.1 | One assay procedures |
| 1.2.1.1 | with fixed time interval |
| 1.2.1.2 | with variable time interval |
| 1.2.2 | Two assay procedures with identical reaction mixture |
| 1.2.2.1 | Reaction started at different times |
| 1.2.2.2 | Reaction stopped at different times |
| 1.2.3 | Two assay procedures with non-identical reaction mixture |
| 1.3 | Multiple point procedures for determining rate of conversion |
| 2. | Continuous data acquisition |
| 2.1 | analogue signal direct output |
| 2.2 | analogue signal handled within system |

In the case of spectrometry the measurement usually leads to a radiant power difference or an absorbance difference (quantity measured: $Q$), which is tranformed into the final result (quantity assessed) for instance catalytic activity concentration, by applying a mathematical function. In two-point procedures two $Q$-values are required.

Some instruments take several readings during very short time intervals (quantity elements: $q_i$) which are transformed into the quantity $Q$. Five examples for the transformation of signals into results are presented in figure 1. The number of quantity elements $q_i$ is mainly of technical consideration and of less importance than the choice of the calculation procedure. The extent of reaction $\xi$ (the notations used throughout this section are in accordance with those given by IUPAC [6]), when a reaction has time-independent stoichiometry is defined as:

$$\xi = \frac{n_i - n_{i,0}}{\nu_i\,(n)} \tag{1}$$

where $n_{i,0}$ is the amount of substance of species i at time = 0 and $n_i$ at any time and where $\nu_i$ (n) is the stoichiometric coefficient of the species i. $\xi$ is independent of the species be it a reactant or a product.

The rate of conversion $\dot{\xi}$ is the time derivative of $\xi$:

$$\dot{\xi} = \frac{d\xi}{d_t} = \frac{1}{\nu_i\,(n)}\,\frac{dn_i}{dt} \tag{2}$$

The rate of reaction $v$ is the rate of conversion divided by volume $V$ of the analytical system; when the volume is constant during the reaction:

$$v = \frac{\dot{\xi}}{V} = \frac{1}{\nu_i V}\,\frac{dn_i}{dt} = \frac{1}{\nu_i\,(n)}\,\frac{dc_i}{dt} \tag{3}$$

where $c_i$ is the amount of substance concentration of species i.

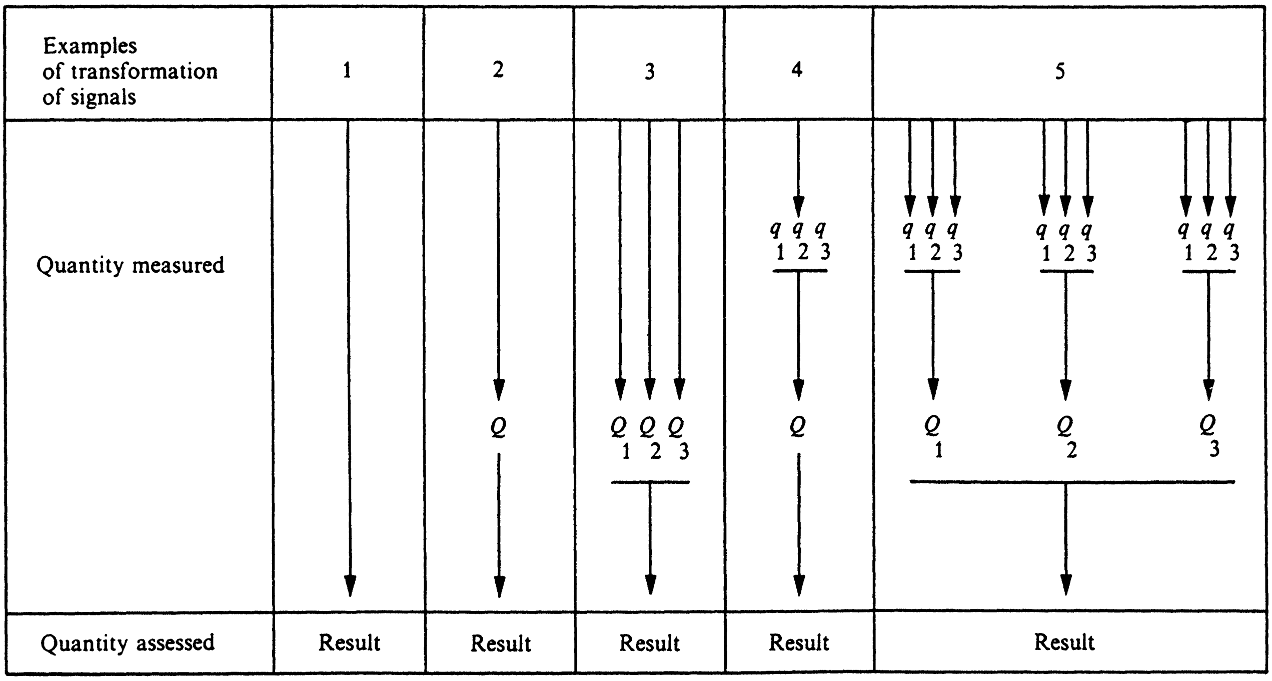

*Figure 1. Transformation of signal to result.*

When the volume of the analytical system changes during the reaction a correction factor must be added to $v$ [6].

Until recently, rate of conversion was termed rate of reaction, contrary to common usage in kinetics, which often follows amount of substance concentration $c$ rather than amount of substance $n$. Thus 'rate of reaction' should now be named 'rate of conversion' according to a recent IUPAC definition [6].

*2.1. Discontinuously measuring methods*

In the simplest procedure, only one measurement of the quantity $Q$ is obtained during the progress of the reaction. The measurement can be made either after stopping the reaction or, if accurately timed, while the reaction is in progress. Such procedures are commonly called 'one point procedures'. To obtain the quantity difference $\Delta Q$, for this approach an additional measurement of the quantity $Q$ has to be made under conditions where the reaction cannot take place. These methods should not be used if the reaction includes a lag phase (*3.2.5*) or if a sample blank occurs.

When the quantity observed, absorbance for instance, is measured twice during the progress of the reaction, the two measurements can be taken either from one assay or from two separate assays.

When the quantity observed is measured at two different times, either the time interval $\Delta t$ (fixed-time) or the quantity difference $\Delta Q$ (variable procedure time) can be kept constant.

From theoretical considerations it can be shown, that the fixed-time approach is to be preferred for first order reaction for the determination of substrate concentration (*5.1.2*). In contrast the variable time procedure is more suitable for the determination of catalytic activity concentration of most enzymes or other catalysts [7].

Some procedures require that measurements be made on two aliquots from the same specimen. These reactions are either started simultaneously, the quantity being measured at two different times, or started at two different times and the quantity measured simultaneously [8]. The reactions may also be started at the same time and be measured after the same interval for both assays if one of the reactions has been omitted in one assay. In each case a single quantity difference between the measurements made on the two assays is used for the calculation of the rate of conversion.

If a lag phase is present, the first measurement should be made only when the rate of conversion has become constant, for example when the absorbance measured is a linear function of time.

In multiple point procedures for determining the rate of conversion, the measured quantity is usually recorded several or many times while the reaction proceeds in one assay. Three measurements ($Q$) must be made to calculate at least two quantity differences.

*2.2 Continuously measuring methods*

By using instruments with analogue data output, continuous recording of the measured quantity is the method of choice for manual procedures [9]. The quantity measured is continuously displayed to the operator, for example on a recorder or an oscilloscope.

### 3. Theoretical aspects of minimal requirements for evaluation of rates of conversion for calculating catalytic activity concentrations of enzymes

*3.1. Current definitions and recommendations by professional societies*

The acceptance by IFCC, IUB and IUPAC [9] of common definitions of terms has been a necessary prerequisite for the production of IFCC methods for the determination of the catalytic activity concentration of enzymes.

*3.1.1.*

Catalytic activity (symbol $z$) of an enzyme is a property quantified by the catalysed rate of conversion of a specified chemical reaction, produced in a specified assay system [10 and 11].

*3.1.2.*

Catalytic activity divided by the volume of the original system from which the samples comes is named catalytic activity concentration (symbol $b$) [9].

*3.1.3.*

It is recommended by IUPAC-IUB [10] that determination of enzyme activity be based on zero-order reaction with respect to substrate. This is often achieved by measuring the initial rate of conversion after exclusion of irregularities of the start reaction.

*3.1.4.*

The unit of catalytic activity is katal (kat), i.e. mol $s^{-1}$. According to its definition, catalytic activity is expressed in the same SI unit as rate of conversion [9].

*3.1.5.*

Catalytic activity concentration has the unit kat $l^{-1}$ or mol $s^{-1}$ $l^{-1}$.

*3.1.6.*

Calculation of catalytic activity concentration using spectrometric methods:

$$b = \frac{\Delta A}{\Delta t} \frac{V}{\varepsilon\, l\, V_s} \text{ or } b = \frac{dA}{dt} \cdot \frac{V}{\varepsilon\, l\, V_s} \quad (4)$$

where:

$\Delta A$ is the absorbance difference (unit: 1)
$b$ is catalytic activity concentration (unit: kat $l^{-1}$)
$l$ is the pathlength of the cuvette (unit: m)
$\Delta t$ is the time difference (unit: s)
$V$ is the volume of the assay system (unit: 1)
$V_s$ is the volume of the sample (unit: 1)
$\varepsilon$ is the molar lineic absorbance (unit: $m^2$ $mol^{-1}$)
$\frac{dA}{dt}$ is the instantaneous slope (unit: $s^{-1}$).

*3.2. Variable rates of enzyme-catalysed reactions due to the mechanism of enzyme catalysis*

In descriptions of methods for assays of enzymes the selection of reaction conditions should support a catalysed rate of conversion which during a reasonable period of time has a constant value, i.e. zero-order kinetics (with respect to substrate). However, there are many reasons why this situation is rarely achieved. These will be discussed in the following sections. The reaction conditions and duration of reaction defined in recommended and selected methods for measurements of catalytic activity of enzymes can minimize, but not totally eliminate, sources of variable values of the rate of conversion.

*3.2.1.*

One-substrate reactions, catalysed by a single enzyme, obey zero-order kinetics provided the concentration of substrate is saturating. Due to accumulation of (inhibitory) products, reverse reaction and depletion of substrate, deviation from zero-order kinetics soon occurs. This results in decreasing values of the measured rate of conversion.

*3.2.2.*

If multiple forms of the enzyme, and not just a single form, act as catalysts, the measured rate of conversion may not assume a constant value. This is due to differences in the catalytic properties of the different forms.

*3.2.3.*

Reaction conditions cannot always be chosen to support a zero-order reaction. It may be necessary to accept non-saturating concentrations of the substrate for instance because of low solubility, inhibitory effect or high absorbance of the substrate.

*3.2.4.*

In two-substrate or multiple-substrate reactions, departure from zero-order may occur for the same reasons as in one-substrate reactions. However, the difficulties of keeping the enzyme saturated with substrate are more pronounced. The frequency of substrate and product inhibition also increases.

*3.2.5.*

Catalytic activity can be determined using techniques in which the primary reaction is coupled to an indicator reaction catalysed by an indicator enzyme. One or more auxiliary reactions may be involved. This may lead to a significant period of non-linearity during the build-up of a steady-state concentration of product(s) from the primary reaction, acting as substrates of the auxiliary and/or indicator reaction(s). This period is called the 'lag phase'. Its duration depends on the kinetic properties of the auxiliary and/or indicator enzyme(s); it can be shortened by using higher catalytic activity concentrations of these enzymes.

In most cases, the steady-state concentration of the substrate of the indicator reaction that is obtained after the lag phase will not fulfill the requirements of zero-order kinetics for the indicator reaction. However, it is stable in the steady state though the concentration is very low. Most of the two-substrate indicator reactions dependent on pyridine coenzyme allow a constant rate of conversion even at relatively low coenzyme concentrations [12]. After the lag phase, a pseudo-zero-order reaction will therefore be obtained.

*3.3. Variable rates of enzyme-catalysed reactions not inherent to the mechanism of catalysis*

*3.3.1.*

Changes in the catalytic properties of the enzyme molecule may occur during the measurement, for instance by:

(*a*) Thermal or chemical inactivation of the enzyme.
(*b*) Unstable pH value of the reaction mixture.
(*c*) Improper thermostating.
(*d*) Removal or inactivation of activators.
(*e*) Introduction of contaminants from instrument parts, acting as modifiers of the catalysed process.
(*f*) Autocatalysis (conversion of proenzymes within the reaction mixture).

*3.3.2.*

Chemical and physical phenomena unrelated to the catalytic properties of the enzyme, for example:

(i) Unstable end-products of the catalysed reaction.
(ii) Reagent-blank reactions (*3.4*).
(iii) Sample-blank reactions (*3.4*).
(iv) Changing turbidity of the analytical system.
(v) Incorrect addition and mixing of reagents.
(vi) Gas bubbles and particles in the analytical system.

*3.4. Blank reactions*

Blank reactions constitute all reactions that are detectable as rate processes by the measuring device and that cause incorrectly determined rates of conversion for the reaction of interest.

Blank rates of conversion may be constant or variable with time. They should, by selection of proper reaction conditions, be low in comparison with the overall rate of conversion. The blank rates should be described in the method together with directions for their compensations when this is necessary to avoid bias. For an example see reference [12]. Two kinds of blank reactions are to be considered (as follows).

*3.4.1.*

Reagent blank reactions affect rates of conversion in the complete analytical system except the sample.

*3.4.2.*

Sample blank reactions are due to one or more components of the sample in the absence of one reaction component, and give rise to rates of conversion other than the one produced by the component to be assessed.

*3.4.3. Compensation for blank reactions*

The magnitude of the effects of blank reactions depends on the reaction conditions on the quality of reagents and on the composition of the sample. The methods of compensation for blank reactions depend on the way in which measurement is made (table 1).

Measurements of blank reactions should be made in a way similar to the one by which the overall catalysed rate of conversion is measured. More particularly, the times at which measurements are performed must be the same. Therefore the lag phase and the time interval during which the blank reaction is followed should be the same as for the overall reaction.

A blank reaction may be measured separately. It is then compensated for arithmetically as part of the calculation of the catalytic activity concentration of the enzyme. It may also be measured simultaneously with the overall reaction and compensated for electronically by the measuring system.

The composition of the reaction mixture for measuring the blank reaction rate depends on the kind of blank reaction to be compensated. A complete reaction mixture without sample is used in compensation for reagent blank reactions. Sample plus an incomplete reaction mixture is used in the compensation for sample blank reactions. Even with both these corrections not all 'false' contributions to the overall rate of conversion are necessarily removed.

### 4. Calculation and validation of the catalytic activity concentration of enzymes

*4.1. Calculation methods*

The mathematical procedures used for converting the measurement quantities to final results are described in the following. All calculations should be performed with quantities measured when the catalysed reaction proceeds as a zero-order or pseudo-zero-order reaction. Only procedures using the fixed-time approach are considered.

*4.1.1. Delta methods*

The difference between the values of a quantity, such as absorbance measured at two different times ($\Delta A$) is the so-called delta. The application of the delta methods requires defined time intervals between successive measurement and use of more than two values, it is usual to work with constant time intervals. The calculation of the catalytic activity concentration of enzymes is based on the deltas. It is possible to estimate to what extent the linear function fits the observed successive quantities by comparing the various deltas with each other, for example by forming ratios or differences; in the last case the time intervals must be identical.

*4.1.2. Regression methods*

By using more than three quantity values ($Q$) it is possible to calculate the slope of the absorbance curve against time by the linear regression method.

The regression procedure may be improved if the quantities measured ($Q$) are obtained at least at five different times. This allows the exclusion of up to two outliers (statistical methods are available for the detection of outliers, for more details see statistical textbooks). For instance with seven values there are 21 combinations of five values resulting in 21 determinations of the slope and 21 accompanying variances. The slope with the lowest variance is the improved slope from which the catalytic activity concentration is calculated [13]. It is recommended to use at least five quantity values to warrant good results, especially if there are outliers. The linear regression method is formulated as:

$$Q = a + bt \qquad (5)$$

where $a$ is the intercept, $b$ is the slope and $t$ the time. The slope has the dimension of $Q/t$ ($A/t$ if absorbance is measured); it is inserted in equation 4 in place of $\Delta A/\Delta t$.

*4.1.3. Derivative methods*

In using derivative methods the quantity function is followed continuously or at (very) small consecutive time deltas. The function is linear if the second derivative is equal to zero. The catalytic activity concentration of enzymes will be calculated from the first derivative. In practice, different methods for calculating and evaluating catalytic activity concentrations of enzymes are applied (for instance the slope can be calculated with the regression method and the linearity can be checked with a delta method).

*4.1.4. Integral methods*

The quantity function is integrated during different time segments. The catalytic activity concentration of enzymes is obtained from the difference between the integrals of the various segments. This method may be applied using electronic (analogue) circuits for continuous integration or microprocessors integrating the digitized signals. The integral method has the advantage that outlying values influence the final result less than methods using discrete values of the function.

*4.2. Evaluation methods*

Evaluation methods should detect those values from the set of quantities measured which could lead to erroneous results.

Common causes are:

(*a*) Non constant conversion rates (*3.2* to *3.4*).
(*b*) Outliers: single value(s) deviating significantly from the linear range of the function.
(*c*) Exhaustion of substrate cofactor.
(*d*) Interfering sample blank reactions (*3.4*).

The first two errors can only be detected using multiple-measurement points. The third error which frequently occurs in the clinical laboratory can be detected by non-linear response. In case of total exhaustion of substrate(s) or cofactors(s) this can be detected by measuring the difference between the absorbance at the beginning and at the end of the reaction. This difference should always be checked when very low results are recorded. Another approach for detecting substrate exhaustion can be measurement at two different wavelengths (bichromatic measurement). The fourth error can be detected and compensated for using two assay procedures with non-identical reaction mixtures (*3.4.3*).

*4.2.1. Visual evaluation*

Errors not easily disclosed by calculation methods may be detected by visual inspection of, for example a plot. This method, therefore, is a valuable alternative or supplement to any computed check of errors.

*4.2.2. Evaluation of multiple-point procedures*

Non-linearity and possible outliers (called 'errors') can only be detected with multiple measurements provided that the measurement intervals chosen are sufficiently large in relation to the resolution of the instrument.

*4.2.3. A procedure to determine confidence limits for calculated results*

A good estimation of the distribution of the errors can be obtained from about 100 results. These results should stem from correctly made measurements showing only random variation.

A tolerable value $\alpha$ for the probability of first kind error – usually $\alpha = 5\%$ – has to be chosen according to the need of the laboratory (this is important because this error implies the number-fraction of false alarms!).

As lower and upper confidence limits the $\alpha/2$ and the $100 - \alpha/2$ per cent quantiles of the above error distribution will be taken.

In the special case where the error variable has a *Gaussian* distribution and $\alpha = 5\%$, the confidence limits for a calculated result r are $r - 2s$ and $r + 2s$ (s = standard deviation).

### 5. Calculation and evaluation methods for the determination of analyte concentrations from conversion rates

*5.1. Theoretical considerations and calculation methods*

Conversion rate procedures for the determination of analyte concentrations can be based on catalysed and non-catalysed reactions. Methods for calculations according to various orders of reaction are described in the following [14].

*5.1.1. Zero-order reactions*

Zero-order reactions are typically applied for calculating the catalytic activity concentration of enzymes. The characteristics for this kind of measurement have already been described in section *3.2.* The technique has been extended to determine the concentration of enzyme activators and inhibitors by measuring their influence on the rate of enzyme-catalysed reactions, for example in the determination of heparin using a chromogenic substrate [15], and in the determination of antibodies or antigens by means of enzyme labelled antigens or antibodies in enzyme-immuno-assay [16 and 17].

If the volume is constant throughout the reaction, zero-order reactions are characterized by:

$$v = -\frac{d[S]}{dt} = k_0 \qquad (6)$$

where:

$k_0$ = rate constant (unit: mol $l^{-1}$ $s^{-1}$)

[S] = substrate concentration (unit: mol $l^{-1}$)

$t$ = time (unit: s)

$v$ = rate of reaction (unit: mol $l^{-1}$ $s^{-1}$).

Because the rate of reaction is independent of the substrate concentration, the zero side cannot be used for the determination of substrate concentration. If the analyte, however, inhibits the activity of the enzyme, a dependency between the concentration of the analyte and the rate of reaction may be found:

$$v = f(c_i) \qquad (7)$$

where:

$c_i$ = concentration of analyte $i$

$v$ = rate of reaction.

Whether the function is linear or non-linear, several calibrators are necessary for calculating the analyte concentration (calibration-curve).

*5.1.2. First-order reactions*

The simplest case of a first-order reaction is

$$S \xrightarrow{k_1} P \qquad (8)$$

where:

S = substrate

P = product

$k_1$ = rate constant (unit: $s^{-1}$).

In this case the rate of reaction is defined as:

$$v = \frac{-d[S]}{dt} = [P]\,k_1 \qquad (9)$$

where:

[S] = substrate concentration (unit: mol $l^{-1}$)

$t$ = time (unit: s)

$v$ = rate of reaction (unit: mol $l^{-1}$ $s^{-1}$).

For enzymatic methods, using the *Michaelis-Menten* expression the following applies [18]

$$v = [S]\,\frac{v_{max}}{[S] + K_m} \qquad (10)$$

where:

$K_m$ = *Michaelis-Menten* constant (unit: mol $l^{-1}$)
$v_{max}$ = maximum rate of reaction.

This implies that the substrate concentration alone must be the limiting factor [19 and 20], when $[S] \ll K_m$. The part of the reaction in which a linear proportionality exists between rate of reaction and substrate concentration and therefore in which an analytical determination of substrate concentration can be based on rate of reaction, is for values of $[S]/K_m$ below 0.2 [20 and 21]. The error in measuring the initial rate can be neglected for $[S]/Km$ <0.05 [22]. Therefore it can be stated, when

$$[S] + K_m \simeq K_m \text{ or when } [S] \ll K_m$$

$$v = [S]\frac{v_{max}}{K_m} \simeq [S]\,k_1 \qquad (11)$$

In order to increase an apparent $K'_m$, competitive inhibitors can be added to the reaction mixture [23]:

$$K'_m = K_m + K_m\frac{[I]}{K_1} \qquad (12)$$

where

$[I]$ = inhibitor concentration

$K_1$ = dissociation constant of the enzyme-inhibitor complex.

Provided that $[S] \ll K'_m$

$$v = [S]\frac{v_{max}}{[S] + (K_m + K_m[I]/K_i)}$$

$$= [S]\frac{v_{max}}{[S] + K'_m} \simeq [S]\,k'_i \qquad (13)$$

The first-order reaction under these conditions is also described by the assumption of *Michaelis-Menten:* The rate-limiting step in an enzyme-catalysed reaction is the turnover of the enzyme-substrate complex to product(s) and enzyme. By integration of equation 9 with respect to time from $t = 0$ to $t$ it follows that

$$[S] = [S]_0\, e^{-kt} \qquad (14)$$

where

$[S]_0$ = initial substrate concentration.

This shows that for first-order reactions there is an exponential relationship between absorbance and time.

If the measured quantity (for example the absorbance difference $\Delta A$ for the interval $t_1$ to $t_2$) is linear with respect to the concentration of substrate or the concentration of product, the initial substrate concentration can be calculated using a first-order approach. Since at time $t$:

$$[P]_t = [S]_0 - [S]_t \qquad (15)$$

where:

$[P]_t$ = product concentration at time $t$

it follows, according to equation (14), that

$$[P]_1 = [S]_0\,(1 - e^{-kt_1});$$

or

$$[P]_2 = [S]_0\,(1 - e^{-kt_2}). \qquad (16)$$

If the absorbance

$$A_1 = \varepsilon[P]_1, \text{ and } A_2 = \varepsilon[P]_2 \qquad (17)$$

it follows that

$$\Delta A = \varepsilon([P]_2 - [P]_1) \text{ and} \qquad (18)$$

$$\Delta A = \varepsilon[S]_0(e^{-kt_1} - e^{-kt_2}) \text{ and} \qquad (19)$$

Therefore

$$[S]_0 = \frac{\Delta A}{\varepsilon(e^{-kt_1} - e^{-kt_2})} \qquad (20)$$

For situations in which absorbance increases with time a simple check for the first-order reaction is to plot $-\ln(A_\infty - A_t)$ versus time, which should be a linear function [14].

In some special cases, the slope of the initial rate of conversion can be used for calculating the analyte concentration [24].

### *5.1.3. Second-order reactions*

In second-order reactions, the analyte concentration is not in a linear relationship to the rate of conversion. The maximum conversion rate (peak rate) can be used for calculation rather than fixed-time reading (25).

### *5.2. Advantages and disadvantages of conversion rate methods for the determination of analyte concentrations*

Advantages of conversion rate methods in comparison to equilibrium methods may be:

Elimination of sample blanks;

Shorter analysis time as compared to equilibrium methods;

Reduction of interferences in cases where the interfering compound leads to a reaction with kinetics different from that caused by the analyte.

The disadvantages of the kinetic approaches described above are larger dependencies on experimental variables such as temperature, pH, inhibitors, activators, etc. and the limited linear range for substrates of enzyme reactions. These problems can be greatly reduced using special multipoint curve-fitting methods [26].

### References

1. Bechtler, G., Haeckel, R., Hørder, M., Küffer, H. and Porth, A. J., *Journal of Antomatic Chemistry*, **23** (1985), 493–503.
2. Harris, R. C. and Hultman, E., Kinetic methods that are independent of the rate of reaction. *Clinical Chemistry*, **29** (1983), 2079–2081.
3. Haeckel, R., Mechanization and automation of analysis. In Bergmeyer, H. U., *Methods of Enzymatic Analysis.* Vol. 1, 3rd edn (Verlag Chemie, Weinheim, 1983), pp. 450–481.
4. Pardue, H. L., A comprehensive classification of kinetic methods of analysis used in clinical chemistry. *Clinical Chemistry*, **23** (1977), 2189–2201.
5. *NCCLS Proposed Standard: PSC-7. Guidelines for Kinetic Analysis of Enzyme Reactions* (1979).
6. *Symbolism and Terminology in Chemical Kinetics. IUPAC document. Pure & Applied Chemistry*, **53** (1981), 753–771.
7. Ingle, J. D. and Crouch, S. R., Theoretical and experimental factors influencing the accuracy of analytical rate measurements. *Analytical Chemistry*, **43** (1971), 697–701.
8. Trayser, K. A. and Seligson, D. A., A new kinetic method for enzyme analysis suitable for automation. *Clinical Chemistry*, **15** (1969), 452–459.
9. Bowers, G. N., Bergmeyer, H. U., Hørder, M. and Moss, D. W., *Approved Recommendations (1978) on IFCC Methods for the Measurement of Catalytic Concentrations of Enzymes. Part 1. Clinical Journal of Clinical Biochemistry*, **18** (1978), 89–95.

10. *Nomenclature Committee of the International Union of Biochemistry: Units of Enzyme Activity, Recommendations 1978. European Journal of Biochemistry,* **97** (1979), 319–320, corrected *104*, 1 (1980).
11. Dybkaer, R., Approved Recommendation (1978), Quantities and Units in Clinical Chemistry, *Clinica Chimica Acta,* **96**, F155–F204; *Journal of Clinical Biochemistry* (1979), 829–835; *Pure Applied Chemistry,* **51** (1979), 2451–2479.
12. Bergmeyer, H. U., Hørder, M., Moss, D. W. and Tietz, N. W., Provisional Recommendations on IFCC Methods for the Measurement of Catalytic Concentrations of Enzymes. Addendum to Part 2. IFCC Method for Alanine Aminotransferase. *Clinica Chimica Acta,* **105** (1980), 147F-172F, *Journal of Clinical Chemistry and Clinical Biochemistry,* **18** (1980), 521–534.
13. Kramer, D. and Porth, A. J., Ein Auswerteverfahren bei enzymkinetischen Messungen. *Aerztl. Lab.*, **20** (1974), 413–417.
14. Bartl, K., Deeg, R. and Ziegenhorn, J., Die Bestimmung von Substratkonzentrationen auf kinetischer Basis. *GIT, Labor-Medizin,* **25** (1981), 89–94.
15. Teien, A. N., Lie, M. and Abildgaard, U., Assay of heparin in plasma using a chromogenic substrate, 413. *Thrombosis Research,* **8** (1976).
16. Wisdom, G. B., Enzyme-immunoassay. *Clinical Chemistry,* **22**, 1243–1255.
17. Oellerich, M. Enzyme immunoassays in clinical chemistry. Present status and trends. *Journal of Clinical Chemistry,* **18** (1976), 197–208.
18. Tiffany, T. O., Jansen, J. M., Burtis, C. A., Overton, J. B. Scott, C. D., Enzymatic kinetic rate and end-point analyses of substrate, by use of a GeMSAEC fast analyzer. *Clinical Chemistry,* **18** (1972), 829—840.
19. Atwood, J. G. and DiCesare, J. L., Making enzymatic methods optimum for measuring compounds with a kinetic analyzer. *Clinical Chemistry,* **21** (1975), 1263–1269.
20. Giuilbault, G. C., *Enzymatic Methods of Analysis* (Pergamon Press, New York, 1970), pp. 114–175.
21. Ingle, J. D. and Crouch, S. R., Theoretical and experimental factors influencing the accuracy of analytical rate measurement. *Analytical Chemistry,* **43** (1971), 697–701.
22. Faust, U., Keller, H. and Becker, J., Diagramm zur Festlegung von Reaktionsparametern für reaktionskinetische Substratbestimmungen. *Journal of Clinical Chemistry and Biochemistry,* **12** (1974), 42.
23. Mueller-Matthesius, R., Ein neues Konzept zur einfachen Durchführung enzymkinetischer Substratbestimmungen. *Journal of Clinical Chemistry Clinical Biochemistry,* **13** (1975), 169–170.
24. Mueller-Matthesius, R., Enzymatische Glucosbestimmung nach der Glucos-Dehydrogenase-Methode. *Journal of Chemistry and Clinical Biochemistry,* **13** (1975), 187–189.
25. Sternberg, J. C., Rate nephelometer for measuring specific protein by immunoprecipitin reactions. *Clinical Chemistry,* **23** (1977), 1456–1464.
26. Hamilton, S. D. and Pardue, H. L., Kinetic methods having a linear range for substrate concentrations that exceed Michaelis-Menten constants. *Clinical Chemistry,* **28** (1982), 2359–2365.

### 7. Key to symbols

| Symbol | Quantity | SI Unit |
|---|---|---|
| $A$ | absorbance | 1 |
| $b$ | catalytic activity-concentration of enzyme | $mol\,l^{-1}\,s^{-1}$; (kat/l) |
| $c$ | concentration of substance | $mol\,l^{-1}$ |
| I | inhibitor | 1 |
| $K$ | dissociation constant | 7 |
| $K_m$ | *Michaelis-Menten* constant | $mol\,l^{-1}$ |
| $k_0$ | zero order rate constant | $mol\,l^{-1}\,s^{-1}$ |
| $k_1$ | first order rate constant | $s^{-1}$ |
| $l$ | pathlength | m |
| $n$ | amount of substance | mol |
| P | product of reaction | 1 |
| $Q$ | quantity measured | unit of $Q$ |
| $q$ | quantity element | unit of $Q$ |
| S | substrate of reaction | 1 |
| $\sigma$ (sigma) | standard deviation | unit of $Q$ |
| $t$ | time | s |
| $V$ | volume of the assay system | 1 |
| $V_{max}$ | maximum rate of reaction | $mol\,l^{-1}\,s^{-1}$ |
| $V_s$ | volume of the sample | 1 |
| $v$ | rate of reaction | $mol\,l^{-1}\,s^{-1}$ |
| $z_E$ | catalytic activity of enzyme | $mol\,s^{-1}$; (kat) |
| $\alpha$ (alpha) | value of probability | 1 |
| $\Delta$ (delta) | difference | 1 |
| $\xi$ *(xi)* | extent of reaction | mol |
| $\dot{\xi}$ | rate of conversion | $mol\,s^{-1}$ |
| $\varepsilon$ | molar absorbance | $m^2\,mol^{-1}$ |
| $\nu$ (n) | stoichiometric coefficient | 1 |

**ATB '87 — ADVANCED TECHNOLOGY FOR THE CLINICAL LABORATORY AND BIOTECHNOLOGY — THIRD EUROPEAN EDITION OF THE OAK RIDGE CONFERENCE**

*26–28 November 1987, Milan, Italy*

ATB '87 is organized by the International Federation of Clinical Chemistry; the Office for Regional Congresses of the IFCC for the African, Mediterranean and Near East Countries; and the Italian Society of Clinical Biochemistry; in co-operation with the American Association for Clinical Chemistry. Papers and posters will review new ideas or new technology relating to the clinical laboratory. Components or systems that can be used in advanced technology will also be considered.

The registration fee is US $70.00.

*Details from ATB '87 Conference, Via C. Farini 70, 20159 Milan, Italy. Tel.: 39 2 6070074 (international), 02 6070074 (national).*

